# Diseases of the Eye

**Published:** 1904-05

**Authors:** 


### BOOK REVIEWS.

Diseases of the Eye. By L. Webster Fox, A. M., M. D., Professor of Ophthalmology in the Medico-Chirurgical College of Philadelphia; Ophthalmic Surgeon in the Medico-Chirurgical Hospital. Octavo, pp. 603, with five colored plates and 296 illustrations in the text. New York and London: D. Appleton & Company. 1904. (Sold by subscription only, price $4.00.)

Works on diseases of the eye have been appearing, of late, with unusual frequency. The list is already long, and it scarcely seems necessary to extend it. Too often these works have been compilations only, and although good as such, they lack that discrimination which experience alone can give. Dr. Fox, however, has offered a work to the profession which is the result of a long and varied experience, both as a teacher and practitioner, and from this point of view his treatise is welcome, and deserves more than a passing notice. It is concisely written, and briefly considers not only the diseases of the eye and their medical and surgical therapeutics, but also the development of this organ and its anatomy and physiology.

Pathology is very sparingly dealt with, and extended details are, for the most part, omitted. The author is thus enabled to give more space to symptomatology, diagnosis and treatment, and these subjects are presented in a careful and thorough manner. The work is amply illustrated, both in black and colors, and many of the illustrations are drawn from the author's own cases and reproduced by means of photo-engraving.

In diagnosis and treatment he avails himself of the latest electrical and *x*-ray appliances, and fully describes them. The newer remedies are also referred to, and their use is appropriately advised. Among these may be mentioned argyrol, dionin, adrenalin, itrol, cuprol, scopalamin and holocain. One of the most admirable features of the work is the prominence the author has given to the various operations on the eye and its appendages. He reviews, quite fully, those which have been proposed by others, and adds those which are original with himself, as well as original modifications of those of others. It is in these sections of the work that the author has made the most substantial contribution to the progress of ophthalmology. Being himself an expert operator, and evidently having quite a genius for devising new methods and improvements, his suggestions and recommendations undoubtedly have much merit, and are worthy of serious consideration by all ophthalmic surgeons. Although much attention has thus been given to operative procedures, yet such important subjects as refraction, accommodation and ocular motility, and those diseases which require medication, alone, have by no means been slighted.

The style of the author is clear, and in drawing from his own experience, his personality becomes apparent throughout the work.

To those who desire the judgment and suggestions of an experienced man in the diagnosis and treatment of diseases of the eye, this work is to be commended. It is not too large and diffuse for the student, it is not too small and abbreviated for the practitioner, and it contains much that will enlighten and stimulate the specialist. A. A. H.

